# Italian validation of the Belastungsfragebogen Parkinson kurzversion (BELA-P-k): a disease-specific questionnaire for evaluation of the subjective perception of quality of life in parkinson’s disease

**DOI:** 10.1186/s40734-017-0059-x

**Published:** 2017-07-25

**Authors:** Paola Ortelli, Roberto Maestri, Marianna Zarucchi, Veronica Cian, Elisa Urso, Francesca Giacomello, Davide Ferrazzoli, Giuseppe Frazzitta

**Affiliations:** 1 Department of Parkinson’s disease, Movement Disorders and Brain Injury Rehabilitation, “Moriggia-Pelascini” Hospital, Via Pelascini, 3, Gravedona ed Uniti, 22015 Como, Italy; 2 Department of Biomedical Engineering, Istituti Clinici Scientifici Maugeri Spa Società Benefit, IRCCS, Via per Montescano 31, Montescano, 27040 Pavia, Italy

**Keywords:** Quality of life, Wellbeing, Rehabilitation treatment

## Abstract

**Background:**

Quality of life (QoL) is the sense of well-being perceived by people. The improvement of parkinsonian patient’s QoL is a crucial goal for clinicians involved in rehabilitative care. In order to provide an appropriate endpoint for the assessment of the effectiveness of rehabilitation treatments on QoL of patients with Parkinson’s Disease (PD), in this study we have first translated and then validated the Belastungsfragebogen Parkinson kurzversion (BELA-P-k). This tool allows evaluating separately two crucial aspects: i) the loss of personal autonomy in activities of daily life and ii) the psychological and psychosocial impact of the disease.

**Methods:**

The BELA-P-k was translated from Dutch into Italian. Subsequently 202 PD patients filled out the questionnaire. Patients were also evaluated by using the Parkinson Disease Questionnaire −39 (PDQ39), the Unified Parkinson’s Disease Rating Scale (UPDRS), the Mini Mental State Examination (MMSE) and the Frontal Assessment Battery (FAB).

**Results:**

The internal consistency for total of two different scores *Bothered by* (Bb) and *Need for Help* (NfH) was excellent (*p* = 0.91) for both categories. The correlation between Bb and NfH categories was significant and strong, very-strong, ranging from 0.78 to 0.88 (all *p* < 0.0001). Finally, the value of Spearman r for the relationship between Bb and NfH items and PDQ 39 values were significant (*p* ≤ 0.003).

**Conclusions:**

In conclusion, we validated the BELA-P-k and demonstrated that it is an appropriate and potentially useful tool for assessing QoL in the management of PD.

**Trials registration:**

This trial was retrospectively registered with ClinicalTrials.gov, NCT03073044.

## Background

Parkinson’s disease (PD) is a chronic and neurodegenerative disorder characterized by motor and non-motor symptoms. An impaired ability to acquire and express automatic actions, rigidity, resting tremor, bradykinesia, hypokinesia and postural instability are the cardinal motor symptoms of the disease. However, other non-motor cognitive, emotional and motivational symptoms, such as dysexecutive syndrome, mood dysregulation disorders, anxiety and behavioural alterations affect PD patients [1, 2].

The interaction between these motor and non-motor symptoms is responsible for a negative impact on patients’ daily life. Indeed, a chronic condition such as PD affects the motor abilities, the cognitive domains and the social functioning, which are features that equally contribute to determine the subjects’ quality of life (QoL) [3–5].

QoL is the sense of well-being perceived by people and it depends not only on the presence or absence of a disease, but also on personal, social and environmental factors [6, 7]. An improvement in patients QoL is generally considered a crucial goal for the clinicians.

As a matter of fact, there is not a cure for PD: dopamine replacement therapy (DRT) represents the gold standard for the medical treatment of PD, but its long-term use side effects (such as dyskinesia, motor fluctuations, dopamine dysregulation syndrome) and its inefficacy on the axial disturbances do not provide patients with a curative treatment. Surgical therapies for PD are now largely widespread, but at the moment, several critical issues regarding its use remain open [8]. Recently, rehabilitation has been highlighted as a feasible, effective and complementary treatment for the management of PD. In this course, several studies have suggested the need for a multidisciplinary and intensive rehabilitative approach in order to achieve better results in PD patients [9–13].

In order to evaluate the effectiveness of a specific rehabilitation treatment for PD, a valid and appropriate assessment of QoL is crucial.

There is a great number of available tools for the assessment of QoL. By using some of them, QoL has been assessed in the general population [14, 15]. Other tools have been specifically designed to assess QoL in specific conditions, such as PD [16, 17]: the most widely adopted tool for the evaluation of QoL in parkinsonian subjects in clinical and research context is the Parkinson Disease Questionnaire −39, PDQ39 [18].

The most important advantage of this questionnaire is the possibility to investigate specific sub-dominions of QoL and specific problems for each sub-dominion. The most important limit is the failure to distinguish two different aspects: the impact of loss of personal autonomy in daily life and the psychological and psychosocial impact of specific disease symptoms. The questionnaire *Belastungsfragebogen Parkinson kurzversion* (BELA-P-k) was developed by the Max- Planck Institute in Munich, as part of a standardized psychological diagnostic routine test for PD patients [19]. This dutch version validates separately the questionnaire, that has the advantage of to evaluate the above-mentioned both aspects [20].

Similar to the PDQ39, the BELA-P-k allows the evaluation of different aspects of the disease, but also the impact of loss of the personal autonomy in daily life and the psychological and psychosocial impact of specific disease symptoms. Thus, we believe that BELA-P-k could be an appropriate tool for evaluating the impact of rehabilitation treatment in QoL of parkinsonian patients.

The present study aims at translating the Bela-P-k for the Italian population and at testing both its internal consistency reliability and its validity.

## Methods

Two hundred and two PD patients hospitalized at the Department of Parkinson’s disease and Brain Injury Rehabilitation of the “Moriggia-Pelascini” Hospital (Gravedona ed Uniti, Italy) were enrolled for the study. The Parkinsonian patients were diagnosed according to the UK Brain Bank criteria [21] and were evaluated by a neurologist with expertise in movement disorders field.

The exclusion criteria were: i) Mini Mental State examination (MMSE) [22] < 24, ii) any focal brain lesion detected in brain imaging studies (CT or MRI) performed in the previous 12 months, iii) other chronic diseases with a known impact in QoL.

The study design and protocol were approved by the local Ethics Committee (*Comitato Etico Interaziendale delle Province di Lecco, Como e Sondrio - Italy)* and were in accordance with the World Medical Association’s code of Ethics (Declaration of Helsinki, 1967). The clinicians explained the study protocol. A written informed consent that was obtained by the patients before their participation in the study. This trial was retrospectively registered with ClinicalTrials.gov, NCT03073044.

### Study instruments and data collection

The patients’ evaluation was performed at admission to the hospital: it included a neurological and neuropsychological examination in order to define the disease stage in accordance with the Hoehn & Yahr (H & Y) classification and assess the following measures: the Unified Parkinson’s Disease Rating Scale (UPDRS), the MMSE and the Frontal Assessment Battery (FAB) [23] the PDQ39 and the Bela-P-k. All patients were assessed in the morning, medication “on”-state, 1 h after they had taken the first dopaminergic drug dose.

The BELA-P-k questionnaire was translated from Deutch into Italian, by using the *translation and back-translation* method and following the guidelines for cross-cultural adaptation of QoL measures defined by Guyatt [24] and by Guillemin and colleagues [25].

The validation was determined by comparing the outcome with the PDQ-39.

The BELA-P-k consists of 19 items grouped into 4 subscales:

1. Achievement capability/physical symptoms (Questions 1–5)
2. Fear/emotional symptoms (Questions 6–9)
3. Social functioning (Questions 10–14)
4. Partner-bonding/family (Questions 15–19).

Each question aims at investigating three specific aspects. First of all, the presence of a certain problem, which is evaluated by a a dichotomous value, that is “yes” or “no”. For every highlighted problem, patient has to describe i) the perceived discomfort related to the problem (“Bothered by” -Bb- and ii) the possible loss of personal autonomy due to that problem (“Need for Help” -NfH-). Both of these aspects are scored on a 5-point Likert scale and permit to obtain two sub-total scores by summing every single question scores.

### Statistics

Clinical data are reported as mean ± SD, while Bela-P-k results are expressed both as mean ± SD and median (lower quartile, upper quartile). Numbers (frequency) are reported for categorical variables. The internal consistency reliability of the Italian version of the Bela-P-k questionnaire was assessed by means of Cronbach’s alpha coefficient. Both the global Bb and the global NfH sub-scale scores of all items (physical symptoms, emotional functioning, social functioning and partner-bonding/family) were tested.

Convergent and discriminant validity were assessed by Multitrait Scaling Analysis [26]. Item convergence was supported if the correlation, corrected for overlap with the trait, that it is hypothesized to represent, was ≥0.4. Item discrimination was supported if the correlation between the item and the trait, that it is hypothesized to represent, was the highest. The discriminant validity was also assessed by the method of known group comparison, testing for differences between mean values for patients grouped according to H & Y stages. This analysis was carried out by a one-way analysis of variance applied to the global Bb and global NfH scores and to all items.

Finally, the relationship between Bela-P-k and the PDQ39 was assessed by Spearman rank correlation coefficient.

The chosen level of statistical significance was 0.05. When appropriate, a false discovery rate was controlled at 5% employing the Benjamini-Hochberg method. All analyses were carried out by using the SAS/STAT statistical package, release 9.2.

## Results

Table 1 reports the demographic and clinical characteristics of patients. The 57% of patients were in Hoehn and Yahr stage, 3, 37% in stage 2 and 6% in stage 4.

**Table 1:** Demographical and clinical data of patients

Variable	N	
Gender (M)	202	105 (52%)
Age (yrs)	202	65.8 ± 9.0
Hoehn and Yahr	185	2.7 ± 0.6
Education (yrs)	202	11.3 ± 4.2
MMSE^a^ [22]	201	28.3 ± 1.6
FAB^b^ [23]	201	14.5 ± 2.6
Total UPDRS	135	39.7 ± 13.1
UPDRS III	135	19.3 ± 6.2
UPDRS II	135	14.2 ± 5.7
UPDRS IV	135	4.2 ± 3.7

The MMSE and FAB scores have been corrected for normative data from Italian population. See reference ^a^[22] for the validation of Italian version of MMSE and reference ^b^[23] for the validation of Italian version of FAB

Descriptive statistics and internal consistency results are reported in Table 2 for all Bb and NfH scores. The Spearman r for the relationship between Bb and NfH scores is also given.

**Table 2:** Descriptive statistics, internal consistency for Bb (Bothered by) and NfH (Need for Help) score and relationship between the both scores

Variable	N	Bb Mean ± SD	Bb Median (Q1, Q3)	Bb int cons	NfH Mean ± SD	NfH Median (Q1, Q3)	NfH Int cons	Spearman r
Total	198	22.1 ± 16.5	19.0 (10.0,31.0)	0.91	19.7 ± 16.0	15.0 (7.0,28.0)	0.91	0.84
Physical Symptoms	201	6.6 ± 4.8	6.00 (3.00,9.25)	0.72	6.0 ± 4.9	5.00 (2.00,9.00)	0.75	0.78
Emotional Symptoms	201	6.0 ± 4.4	5.00 (2.00,9.00)	0.74	5.1 ± 4.3	4.00 (2.00,8.00)	0.76	0.80
Social Support	201	5.0 ± 5.2	4.00 (1.00,8.00)	0.79	4.6 ± 5.0	3.00 (0.50,7.00)	0.80	0.88
Partner Bonding/Family	198	4.5 ± 4.8	3.00 (1.00,7.00)	0.71	4.1 ± 4.7	2.00 (0.00,6.00)	0.73	0.88

The internal consistency was acceptable (>0.70) for all items of Bb and NfH category scores. A tendency towards values in NfH higher than in Bb was observed with regards to all items. The internal consistency for total Bb and NfH was 0.91 for both categories. The correlation between Bb and NfH categories was significant and strong very-strong, ranging from 0.78 to 0.88 (all *p* < 0.0001).

Table 3 shows the percentage of patients who judged each question as relevant. The most endorsed question was Q1: 80% of patients who thought the problem was relevant to them, with percentages very similar in males and females and with a greater frequency as HY increases, but without reaching statistical significance (Table 4). On the contrary, the least endorsed question was Q18 (28%), with significantly higher occurrences in males (36 vs 19%, adjusted *p* = 0.027). Significant differences between gender were observed also for Q9, Q13, Q16 and Q19 (adjusted *p* = 0.004, *p* = 0.045, *p* = 0.004, *p* < 0.0001, respectively) and between HY stages for Q5 and Q14 (adjusted *p* = 0.006 and *p* = 0.036, respectively).

**Table 3:** Percentage of “relevant judgment” to each question for patients

Variable	presence (%)	M presence (%)	F presence (%)	HY 2 presence (%)	HY 3 presence (%)	HY 4 presence (%)
PS1q	162 (80%)	86 (82%)	76 (78%)	51 (75%)	86 (81%)	10 (91%)
PS2q	131 (65%)	64 (61%)	67 (69%)	40 (59%)	72 (68%)	7 (64%)
PS3q	107 (53%)	55 (52%)	52 (54%)	30 (44%)	58 (55%)	10 (91%)
PS4q	112 (55%)	60 (57%)	52 (54%)	39 (57%)	56 (53%)	8 (73%)
PS5q	107 (53%)	53 (50%)	54 (56%)	22 (32%)	65 (61%)	8 (73%)
ES6q	128 (63%)	69 (66%)	59 (61%)	43 (63%)	69 (65%)	6 (55%)
ES7q	106 (52%)	60 (57%)	46 (47%)	38 (56%)	54 (51%)	5 (45%)
ES8q	147 (73%)	73 (70%)	74 (76%)	53 (78%)	76 (72%)	9 (82%)
ES9q	117 (58%)	52 (50%)	65 (67%)	42 (62%)	59 (56%)	7 (64%)
SF10q	113 (56%)	66 (63%)	47 (48%)	38 (56%)	57 (54%)	7 (64%)
SF11q	90 (45%)	52 (50%)	38 (39%)	30 (44%)	45 (42%)	6 (55%)
SF12q	70 (35%)	39 (37%)	31 (32%)	22 (32%)	37 (35%)	6 (55%)
SF13q	98 (49%)	63 (60%)	35 (36%)	30 (44%)	48 (45%)	8 (73%)
SF14q	79 (39%)	44 (42%)	35 (36%)	15 (22%)	50 (47%)	4 (36%)
PB-F15q	103 (51%)	61 (58%)	42 (44%)	31 (46%)	56 (53%)	8 (73%)
PB-F16q	64 (32%)	45 (43%)	19 (20%)	12 (18%)	39 (37%)	5 (45%)
PB-F17q	74 (37%)	32 (30%)	42 (44%)	24 (35%)	40 (38%)	4 (36%)
PB-F18q	56 (28%)	38 (36%)	18 (19%)	23 (34%)	27 (26%)	4 (36%)
PB-F19q	85 (43%)	68 (65%)	17 (18%)	28 (41%)	44 (42%)	6 (55%)

*PS* Physical Symptoms, *ES* Emotional symptoms, *SF* Social Functioning, *PB-F* Partner-Bonding\Family, *q* question

**Table 4:** Discriminant validity to Bb (Bothered by) and NfH (Need for Help) scores for patients grouped according to H%Y stages

Variable	HY 2	HY 3	HY 4	p val ANOVA
Total_Bb	21.0 ± 17.0	22.3 ± 16.1	29.1 ± 18.9	0.33
Physical Symptoms_Bb	5.8 ± 4.7	6.7 ± 4.7	9.4 ± 4.8	0.056
Emotional Symptoms_Bb	6.1 ± 4.3	5.9 ± 4.5	6.7 ± 4.9	0.84
Social Functioning_Bb	4.6 ± 5.3	5.2 ± 5.2	6.5 ± 5.5	0.55
Partner Bonding\Family_Bb	4.3 ± 4.8	4.6 ± 4.8	6.5 ± 5.6	0.38
Total_NfH	18.3 ± 17.4	19.7 ± 15.0	28.1 ± 18.1	0.18
Physical Symptoms_NfH	5.2 ± 5.0	6.0 ± 4.7	9.5 ± 4.5	0.022
Emotional Symptoms_NfH	5.1 ± 4.2	5.0 ± 4.2	6.9 ± 5.4	0.38
Social Functioning_NfH	4.1 ± 5.3	4.6 ± 4.7	5.8 ± 5.8	0.56
Partner Bonding\Family_NfH	3.9 ± 4.9	4.1 ± 4.6	5.9 ± 5.4	0.42

Item convergence and discrimination were supported for all items of both Bb and NfH category scores (corrected correlation with the trait hypothesized to represent ranging from 0.46 to 0.78 for Bb category scores and from 0.46 to 0.82 for NfH category scores).

The Italian version of Bela-P-k scores showed discrimination between patients with different disease severity according to the H & Y stage, only for the Achievement capability/physical symptoms with a borderline significance (*p* = 0.056) for Bb category and a clear significance (*p* = 0.022) for NfH category.

Table 5 reports the value of Spearman r for the relationship between Bb and NfH items and PDQ 39 values (total score and 3 sub-scores: mobility; well being and social support). All associations were significant (*p* ≤ 0.003).

**Table 5:** Relationship between Bela-p-K and PDQ39

	PDQ_39	PDQ Mobility	PDQ Emotional Well-being	PDQ Social Support
Total_Bb	0.63	0.49	0.59	0.51
Physical Symptoms_Bb	0.62	0.55	0.47	0.39
Emotional Symptoms_Bb	0.56	0.40	0.66	0.44
Social Functioning_Bb	0.52	0.38	0.50	0.45
Partner Bonding\Family_Bb	0.43	0.31	0.38	0.48
Total_NfH	0.54	0.46	0.48	0.33
Physical Symptoms_NfH	0.58	0.56	0.40	0.27
Emotional Symptoms_NfH	0.49	0.38	0.56	0.30
Social Functioning_NfH	0.46	0.36	0.44	0.33
Partner Bonding\Family_NfH	0.35	0.26	0.31	0.36

*Bb* Bothered by, *NfH* Need for Help

## Discussion

A multidisciplinary rehabilitation treatment helps the patients to reduce both the functional impact and the discomfort due to the disease symptoms in everyday living activities and allows patients to acquire strategies to face with their motor difficulties [2, 12, 25, 27].

QoL is a crucial endpoint in the evaluation of the effectiveness of any therapeutic approach, since it explores the personal well-being perceived by patients.

Even if the PDQ39 is a specific and widely used tool for the assessment of QoL in parkinsonian patients, this instrument does not allow to distinguish between the impact of loss of personal autonomy in activities of daily life and the psychological and psychosocial impact of specific disease symptoms.

This is an important distinction in the rehabilitation field, since it permits to evaluate both the functional impact of the symptoms on everyday living and the perceived discomfort.

In this context, we have translated and validated the Bela-P-k. This questionnaire, which requires a short period of administration, permits to obtain two different scores: “Bothered by” (Bb) and “Need for Help” (NfH).

In accordance with previous studies [20], our data indicate that the internal consistency of Bela-P-k is excellent for both total scores of Bb and NfH and is also acceptable for the both scores in each singular question. The correlation between Bb and NfH scores was strong, but it never corresponded to 1.0. For this reason we confirm the advantage in assessing both issues separately.

There was a positive correlation between the QoL assessment obtained by Bela-P-k and PDQ39: this confirms that Bela-P-k is a good tool for clinicians.

In parkinsonian patients, the interindividual differences are often quite striking. In order to evaluate which symptoms are most common, we analysed the frequencies of relevance for each item. The most frequent issue reported by patients is the loss of efficiency in daily living: 80% of parkinsonian people complain about this, with a greater incidence in the advanced stage of the disease, as highlighted in precedent studies [28, 29]. People in the more advanced stages reported more frequently problems in requiring caregiver’s assistance in daily living activities and the need of delegating their duties to others.

We found no gender differences in QoL. However, between the males and females some differences were detected: while a greater fear for the future was found in women, males show a greater loss of identity, a greater influence of the family in their daily lives and sexual difficulties.

We suggest that these differences are due to the different tendency between female and male: the first tends to develop affective symptoms while the males tend to develop behavioural symptoms [30].

## Conclusions

In conclusion, we found that the BELA-P-k is a suitable, valid and easily administrable test. This questionnaire provides the clinicians with an instrument that make possible to distinguish between the specific life’s aspects (Achievement skills, emotional status, social functioning and integration and quality of relationship with partner and relatives) and the components that worsen the Qol (the loss of personal autonomy in daily life and the perceived discomfort of each symptom). Given these features, BELA-P-k could represent a good outcome in a rehabilitative care context, allowing the clinicians to tailor specific rehabilitation treatments.

## Abbreviations

Bb: Bothered by
BELA-p-K: Belastungsfragebogen Parkinson kurzversion
CT: Computed tomography
DRT: Dopamine replacement therapy
FAB: Frontal Assessment Battery
MMSE: Mini Mental State Examination
MRI: Magnetic resonance imaging
NfH: Need for help
PD: Parkinson’s Disease
PDQ-39: Parkinson Disease Questionnaire −39
QoL: Quality of life
UPDRS: Unified Parkinson’s Disease Rating Scale
